# 

**Published:** 1822-03-01

**Authors:** 


### Contents

OF

### No. 8, Vol. II, for MARCH, 1822.

Art. I.

ARACHNOID INFLAMMATION.

Researches on Inflammation of the Arachnoid Membrane, Cerebral and Spinal. By Drs. Parent-Duchatelet and Martinet - - - - - - - - - - - - - - - 695

II.

Essay on the Fucus Helminthocorton, or Corsican Moss, in Cancer. By William Farr - - - - - - - - - - 732

III.

A Series of Lectures on modern Surgery, principally from Sir Astley Cooper. By Charles Myngay Syder - - - 736

IV.

Present State of Medicine in Italy, principally from the writings of Tommasini - - - - - - - - - - - - 745

V.

Observations on those Diseases of Females which are attended by Discharges. By Charles Mansfield Clarke - - 757

VI.

Mr. Stevenson on the Nature and Treatment of Amaurosis 778

VII.

A Treatise on Diseases of the Chest, translated from the French of M. Laennec, by Dr. John Forbes - - - - - - 795

VIII.

Mr. Charles Bell's Experiments on the Respiratory Nerves 809

IX.

Mr. O'Halloran on the Yellow Fever of Andalusia, during the Epidemic of 1820 - - - - - - - - - - - - - 817

X.

Mr. J. H. James on the Principles, Nature, and Treatment of the different Species of Inflammation - - - - - - - 834

XI.

Mr. Henry Earle on the Re-establishment of a Canal in the Urethra - - - - - - - - - - - - - - - - - - 858

XII.

Dr. A. P. W. Philip on Indigestion, *(Second Edition)* - - 862

XIII.

Medico-Chirurgical Transactions, Vol. II. Part 2 - - -

Art. 1. Mr. Hutchison on Bronchocele - - - - - - 866
—— 2. Dr. Gregory on Marasmus - - - - - - - 868
—— 3. Mr. Coates on Fractured Pelvis - - - - - - 870
—— 4. Mr. Price's Case of Sudden Death - - - - - ib.
—— 5. Mr. Coates's Case of Aneurism - - - - - - ib.
—— 6. Dr. Gregory's Case of Malformation - - - 871
—— 7. ———— Case of Chorea - - - - - - ib.
—— 8. Mr. Breton on Pomegranate Bark - - - - 872
—— 9. ———— on Swietenia Febrifuga - - - ib.
——10. Mr. Swan on the Ear - - - - - - - - - 873
——11. Mr. Dunn on Amputation - - - - - - - ib.
——12. Sir Astley Cooper on Extraction of Calculi by a new Instrument - - - - - - - - - 874
——13. Mr. Wilbank on Sloughing Phagedena - - - 875
——14. Mr. Burmester's Case of Tetanus - - - - 876
——15. Mr. Scott's Case of Parturition, with separation of the Os Uteri - - - - - - - - - - ib.
——16. Mr. Salmon's Case of Aneurism - - - - - - ib.
——17. Mr. Martineau on Lithotomy - - - - - - 877
——18. Mr. Porter on Cynanche Laryngea - - - - 878
——19. Sir Astley Cooper's Case of Tumour - - - 880

XIV.

Opiologia; or Confessions of Opium-Eaters, with Practical Remarks on Opium - - - - - - - - - - - - - 881

XV.

M. Fallot on Hydrocephalus - - - - - - - - - - 902

XVI.

Medical Intelligence - - - - - - - - - - - - - 904

XVII.

Correspondence - - - - - - - - - - - - - - - 905

XVIII.

*Extra Limites.*—Dr. Emerson's Medical Botany - - - 906

XIX.

Bibliographical Record - - - - - - - - - - - - 907

XX.

Subscribers, &c. &c. &c. - - - - - - - - - - - 913

